# Polycystins and mechanotransduction in bone

**DOI:** 10.18632/oncotarget.22421

**Published:** 2017-11-13

**Authors:** Antonios N. Gargalionis, Efthimia K. Basdra, Athanasios G. Papavassiliou

**Affiliations:** Athanasios G. Papavassiliou: Department of Biological Chemistry, Medical School, National and Kapodistrian University of Athens, Athens, Greece

**Keywords:** polycystins, mechanotransduction, bone, osteoblast, Runx2

Accumulating evidence suggests that a distinct family of proteins termed polycystins (polycystin-1, PC1 and polycystin-2, PC2) function as important regulators of mechanosensing, mechanotransduction and mechanoresponses in osteoblastic cells affecting their proliferation, differentiation and ultimately bone remodeling [1].

PC1 functions as an atypical G protein-coupled receptor with a long, antenna-like, extracellular end that senses mechanical cues and PC2 functions as a Ca^2+^-permeable channel. Polycystins are expressed in osteoblasts and form circulatory networks with other mechanosensitive structures / proteins such as primary cilia and integrins, respectively. These networks integrate multiple signaling pathways and modulate osteoblastogenesis, adipogenesis and bone mass [1]. However, the molecular mechanisms that govern these processes and the role of polycystins in such settings remain obscure.

It is known that PC1 activates the Janus kinase (JAK)-signal transducer and activator of transcription (STAT) pathway in renal cells, a pathway that is also implicated in bone metabolism. Accordingly, we investigated whether PC1 mediates mechanical stretching via activation of the JAK-STAT signaling cascade in order to induce expression of the osteoblast-specific Runt-related transcription factor 2 (Runx2) in our *in vitro* model of primary osteoblast-like cells. Our data demonstrated that mechanical strain was associated with increased phosphorylation(p)/activation of JAK2 that was PC1 dependent. Duolink antibody-based proximity ligation assay revealed an augmented interaction between PC1 C-terminal tail (CTT) and pJAK2 following mechanical stretching. Furthermore, a quick nuclear translocation of pSTAT3 was detected and STAT3 DNA-binding activity was rapidly increased after mechanical stimulation depending on PC1. Elevated *runx2* mRNA levels were also observed under stretching and pre-treatment with either PC1, JAK2, STAT3 inhibitors abolished Runx2 mRNA upregulation. Chip assays revealed that inhibition of PC1 and JAK2 reduced STAT3 and Runx2 binding to *runx2* promoter, indicating a PC1-dependent regulation of *runx2* gene expression by pSTAT3 and Runx2 transcription factors. Conclusively, a novel network was identified unraveling a PC1-JAK2-STAT3 axis that targets *runx2* gene under mechanical loading. [2].

Our study unveils a novel PC1-dependent pathway that regulates osteoblast differentiation via JAK-STAT cascade and for the first time a direct interaction between PC1 and JAK2 in osteoblasts, therefore further elucidating the role of JAK2-STAT3 pathway in bone homeostasis. These data are in concert with similar studies documenting the role of polycystins as sensors of extracellular mechanical tension in osteoblasts and PC1-dependent Runx2 expression. Stable Polycystic Kidney Disease 1 (*PKD1*, the gene encoding PC1) knockdown in the mouse osteoblastic cell line MC3T3-E1 results in diminished expression of osteoblastic differentiation markers, among them Runx2.

PC2 senses extracellular mechanical signals in osteoblasts, induces increase of intracellular Ca^2+^, phosphorylation of Akt, subsequent phosphorylation of glycogen synthase kinase 3beta (GSK3β) that inhibits *β*-catenin deactivation, thus leading to *β*-catenin translocation to the nucleus and promoting osteoblastic gene transcription (e.g. *runx2*) [3]. In corroboration, *PKD2* (the gene encoding PC2) conditional inactivation in mature osteoblasts in mice has been associated with osteopenia. *PKD2*-deficient mice also present reduction of expression in several mRNAs of osteoblast-specific genes and decrease in adipocyte-specific markers. It seems that PC1 and PC2 functions are not totally alike. PC1 effects are more striking implying that is the chief molecule and PC1 deficiency causes increased adipogenesis and bone marrow fat. PC1 and PC2 act as mechanosensitive molecules along with mechano-induced co-activator TAZ to regulate *runx2* expression (leading to osteoblastogenesis by activating it) and peroxisome proliferator-activated receptor gamma (PPAR-γ) (leading to adipogenesis by repressing it) [4].

Our research group has demonstrated previously the role of PC1 as a principal mechanosensing molecule in primary osteoblast-like cells that induces *runx2* expression via the calcineurin-nuclear factor of activated T-cells (NFAT) axis under mechanical strain [5]. We have also documented that continuous application of hydrostatic pressure in chondrogenic ATDC5 cells controls *runx2* expression and increases *PKD1* and *PKD2* mRNA levels. The expression profile of PC1 follows that of Runx2 confirming that PC1 may regulate *runx2* expression under physical stimulation [6]. Notably, polycystins have been also implicated in molecular mechanisms in human osteoblast-like MG-63 cells, an *in vitro* model for the investigation of osteosarcoma, the most common bone-derived tumor. Stable *PKD1* knockdown in MG-63 cells is associated with increase of cell proliferation, reduced mRNA expression of Runx2 and Osterix, increased adipogenesis and lower Ca^2+^ influx rise as a response to fluid flow [7]. Osteosarcoma in particular displays impaired mechanotransduction and deregulation of mechanosensitive molecules with altered focal adhesion status through activation of mechanosensors of the focal adhesion complex. Preliminary data of our team show that PC1 functional inhibition impairs proliferation and migration in MG-63 cells and activates intracellular oncogenic signaling (mammalian target of rapamycin (mTOR) pathway) [8].

The emergence of polycystins as crucial mechano-regulators of osteoblast differentiation offers new routes for potential development of drugs that could be assessed in preclinical and clinical models of bone loss maladies, osteoporosis, healing of bone-tissue injury (fractures) and in other diseases where polycystins have lately been involved (e.g. colorectal cancer). Such agents may activate mechanosensors potentially mimicking mechanical loading or function as agonists or inhibitors. Nevertheless, further experimental studies should focus on deciphering the molecular mechanisms via which polycystins integrate extracellular mechanical cues and modulate differentiation of osteoblasts hence bone remodeling.
